# Metastatic progression and gene expression between breast cancer cell lines from African American and Caucasian women

**DOI:** 10.1186/1477-3163-6-8

**Published:** 2007-05-01

**Authors:** Haile F Yancy, Jacquline A Mason, Sharla Peters, Charles E Thompson, George K Littleton, Marti Jett, Agnes A Day

**Affiliations:** 1 Department of Arts and Sciences, Coppin State University, Baltimore, MD, 21216, USA; 2 Department of Microbiology, College of Medicine, Howard University, Washington, D.C. 20059, USA; 3 Department of Physiology, Howard University, Washington, D.C. 20059, USA; 4 Division of Pathology, Walter Reed Army Institute for Research, Silver Spring, MD 20910, USA

## Abstract

African American (AA) women have a lower overall incidence of breast cancer than do Caucasian (CAU) women, but a higher overall mortality. Little is known as to why the incidence of breast cancer is lower yet mortality is higher in AA women. Many studies speculate that this is only a socio-economical problem. This investigation suggests the possibility that molecular mechanisms contribute to the increased mortality of AA women with breast cancer. This study investigates the expression of 14 genes which have been shown to play a role in cancer metastasis. Cell lines derived from AA and CAU patients were analyzed to demonstrate alterations in the transcription of genes known to be involved in cancer and the metastatic process. Total RNA was isolated from cell lines and analyzed by RT-PCR analysis. Differential expression of the 14 targeted genes between a spectrum model (6 breast cancer cell lines and 2 non-cancer breast cell lines) and a metastasis model (12 metastatic breast cancer cell lines) were demonstrated. Additionally, an *in vitro *comparison of the expression established differences in 5 of the 14 biomarker genes between African American and Caucasian breast cell lines. Results from this study indicates that altered expression of the genes Atp1b1, CARD 10, KLF4, Spint2, and Acly may play a role in the aggressive phenotype seen in breast cancer in African American women.

## Background

Cancer is characterized by excessive growth and spread of abnormal cells. It affects all populations in the United States and ranks second only to heart disease as the leading cause of death [1]. More than half the recognized types of cancer share the property of metastatic activity [2]. It is estimated that 1,399,790 new cases of cancer will be diagnosed in 2006. More than 564,830 Americans are expected to die of cancer, equaling more than 1,500 people each day. African American (AA) women exhibit a disproportionate burden of cancer. The American Cancer Society reported that in 2005 more than 63,000 AA were expected to die from cancer [3]. For all cancers, cancer death rates among AA are higher than other racial or ethnic populations in the United States [4]. In 2005, it was estimated that 137,910 new cases of cancers would be diagnosed among AA women. Among AA women, the most common cancers will be breast, colon and rectum, and lung. Cancers among AA women are more frequently diagnosed after the cancer has metastasized and spread to regional or distant sites [3]. In 2006 more than 212,920 new cases of invasive breast cancer will be diagnosed and 41,430 women are expected to die due to the disease [1]. Although the 5-year survival rate among AA women diagnosed with breast cancer has improved, they still have a decreased likelihood of surviving 5 years after diagnosis than Caucasian (CAU) for all cancer sites and at all stages of diagnosis. Much of this difference is believed to be due to factors associated with poverty [5], which include reduced access to medical care [6], diagnoses at a later stage, when the disease has spread to regional or distant tissues [7], and disparities in treatment [8,9]. The goal of this study is to identify biological factors that may lead to or increase the high mortality rate observed in AA.

Metastasis is the main cause of morbidity and mortality in cancer patients. The selective distribution of metastases is dictated by numerous factors, including complementary adhesive contacts, the pattern of vascular flow from the primary site, and molecular interactions between the tumor cell and the stroma at the secondary site [10]. Our lab has previously shown that of the 26 human matrix metalloproteinases (MMPs), 12 have been shown to have elevated expression in AA breast cancer cell lines when compared to their CAU counterparts. Our results suggested that there is altered expression of 12 MMPs in cell lines derived from AA and CAU women. The data demonstrated elevated expression of MMPs 3, 7, 8, 9, 11–15, 23B, 26, and 28 in AA women [11]. This investigation indicated that altered expression of MMPs may play a role in the aggressive phenotype seen in AA women. As a result of the aforementioned study, an expanded gene list of possible biomarkers that may be responsible for the aggressive breast cancer observed in the AA women were examined. The experiments were modeled using 14 of the 43 genes described in the study by Eckhardt et al. [12] to create primers using the human analog gene sequences. This study investigates the expression levels of 14 genes, which have been shown to play a role in cancer and the metastatic process, using breast cell lines derived from AA and CAU women.

## Methods

### Cell Culture

Cell lines were purchased from American Type Culture Collection (Rockville, MD, USA) and Coriell Cell Repositories (Camden, NJ). Cells were propagated in the recommended media and given new media every 2 to 3 days until 90% confluent. The spectrum model (Table 1) contains 6 breast cancer cell lines and 2 non-cancer breast cell lines. Of the 6 cell lines, three are derived from AA and three derived from CAU. This model also contains two cell lines from primary sites and one metastatic cell line. The metastatic model (Table 2) consists of 12 metastatic breast cancer cell lines from 6 AA and 6 CAU women.

**Table 1:** Spectrum Model Cell Lines

	**CAUCASIAN (CAU)**
	
**MCF-12A**	**Mammary gland; breast; epithelial; non-tumorigenic**
HS578T	Mammary gland; breast; carcinoma
MCF-7	Mammary gland; breast; epithelial; metastatic site: pleural effusion adenocarcinoma
CRL-2336	Mammary gland epithelial, primary ductal carcinoma

	**AFRICAN AMERICAN (AA)**

AG11132	Mammary gland; breast; epithelial; non-tumorigenic
CRL-2315	Breast, primary ductal carcinoma
CRL-2329	Carcinoma, ductal, primary; breast; mammary gland
CRL-2320	Carcinoma, ductal, breast; mammary gland; from metastatic site: lymph node

**Table 2:** Metastatic Model Cell Lines

	**CAUCASIAN (CAU)**
	
**MCF-12A**	**Mammary gland; breast; epithelial; non-tumorigenic**
CAMA-1	Mammary gland; metastatic site: pleural effusion adenocarcinoma
CRL-2351	Mammary gland; metastatic site: malignant pleural effusion adenocarcinoma
HTB-30	Mammary gland; metastatic site: pleural effusion adenocarcinoma
CRL-2327	Mammary gland; metastatic site: adenocarcinoma and pleural effusion cells adenocarcinoma
MCF-7	Mammary gland; breast; epithelial; metastatic site: pleural effusion adenocarcinoma
HTB-27	Mammary gland; metastatic site: brain adenocarcinoma

	**AFRICAN AMERICAN (AA)**

AG11132	Mammary gland; breast; epithelial; non-tumorigenic
CRL-7721	Mammary gland; metastatic site: pleural effusion carcinoma
CRL-1504	Mammary gland; metastatic site: ascites ductal carcinoma
CRL-2330	Mammary gland: primary metaplastic carcinoma lymph node metastasis
CRL-2320	Carcinoma, ductal, breast; mammary gland; from metastatic site: lymph node
HTB-24	Mammary gland; metastatic site: pleural effusion ductal carcinoma
HTB-132	Mammary gland: metastatic adenocarcinoma of the breast
CRL-2335	Mammary gland: metastatic adenocarcinoma of the breast

### RNA Extractions

RNA was extracted from the cell lines using RNAqueous (Ambion, Austin, TX). Cells were collected by low speed centrifugation and lysed by adding 200 μl of Lysis/Binding Solution. An equal volume of 64% ethanol was added to the lysate. The lysate/ethanol mixture was transferred to the RNAqueous Filter Cartridge and centrifuged for 1 minute at 13,400 rpm. The flow through was discarded and 700 μl of Wash Solution 1 was added to the RNAqueous Filter Cartridge and centrifuged for 1 minute. The column was washed twice with 500 μl of Wash Solution 2/3 and eluted with 110 μl Elution Solution. Isolated RNA was quantitated using the NanoDrop Spectrophotometer (Wilmington, DE). All RNA samples were stored at -70°C in RNA elution solution (Ambion, Austin, TX).

### Reverse Transcriptase Polymerase Chain Reaction (RT-PCR)

The RT-PCR reactions were performed in a P/E GeneAmp 9700 thermocycler (Perkin-Elmer Co., Norwalk CT), using the Access RT-PCR system (Promega, Madison, WI). The reaction mixes were prepared by combining 27.5 μl of nuclease free water, 10 μl of AMV, 1 μl Tfl 5X reaction buffer, 2 μl dNTP mix, 50 pM of upstream primer, 50 pM of downstream primer in 1.5 μl volume each, 3 μl 25 mM MgSO4, 1.0 μl AMV reverse transcriptase, Tfl DNA polymerase and 1 μg of total RNA in a 0.5 ml thin walled Eppendorf tube on ice. The reaction mixes were then vortexed for 5 seconds and centrifuged. The PCR cycling profile was as follows: 48°C for 1 minute for reverse transcription of the RNA into cDNA, 94°C for 4 minutes to deactivate the reverse transcriptase, and 30 cycling sequences of denaturing at 94°C for 45 seconds, annealing at 55°C-58°C (Table 3) for 30 seconds, and extension at 72°C for 1 minute with a final extension at 72°C for 10 minutes. An aliquot of 20 μl of each RT-PCR reaction was run on 1.2% agarose gels, stained with ethidium bromide, photographed and subjected to densitometic measurements using the Chemi-Imager Tm 4000 (Alpha Innotech, Corporation, San Leandro, CA). The primers used in this study are listed in Table 3.

**Table 3:** Gene List

Gene	Accession Number	Symbol	Primer Sequence	Annealing Temperature °C	Base pair size (bp)
ATP citrate lyase	AW538652	Acly	CATCCACAGGCTAACACC CATCCTAACGCCCTACAA	58	179
ATPase, Na+/K+ transporting, b1 polypeptide	AW544616	Atp1b1	CTCTTGCCTTGTCCTCCG CAGCATGTGATGCCTCCA	58	145
Caspase recruitment domain family, member 10	BG085048	Card10	TGAGCCTTCCTAGACCCTT TGCCCATGAGAACTTGAGTG	58	275
Epoxide hydrolase 1, microsomal	BG072453	Ephx1	CCAAGCCTGACACCGTAG GCCAGTGGGCACATAGAC	58	278
UDP-N-acetyl-a-D-galactosamine/polypeptide	BG068045	Galnt3	AGCGTTGGTCAGCCTCTA GTTGTGCCGAATTTCATG	58	135
Kruppel-like factor 4 (gut)	BG069413	Klf4	CCAGCCAGAAAGCACTAC GACTCACCAAGCACCATC	55	409
Peptidylprolyl isomerase C	BG065249	Ppic	TCGGCTGCTGCTACCTCT CTGCCAACATCTTTGTCTCC	55	144
Serine protease inhibitor, Kunitz type 2	BG085206	Spint2	CTTGGCTCAAAGGTGGTG CAAATCCGAGTCAATCCC	55	263
Protein tyrosine phosphatase, receptor type F	BG088014	Ptprf	TCTGCTTCAAACCCTCAA TCTGCTTCAAACCCTCAA	55	136
Transforming growth factor b2	BG067564	Tgfb2	ATTGCCCTCCTACAGACT GTATCCATTTCCACCCTA	55	152
Tissue inhibitor of Metalloproteinase 1	NM_003254	TIMP1	ACAACCGCAGCGAGGAGT AGGTGACGGGACTGGAAGC	55	262
Tissue inhibitor of Metalloproteinase 2	NM_003255	TIMP2	TTGACCCAGAGTGGAACG ACCAAAGACGGGAGACGA	55	101
Tissue inhibitor of Metalloproteinase 3	NM_000362	TIMP3	GTTGTAGGGTTTCTGTTGT GTGTTGTCTGCTGCTTTT	57	310
Tissue inhibitor of Metalloproteinase 4	NM_003256	TIMP4	TACCAGGCTCAGCATTAT CCACTTGGCACTTCTTATT	55	232
Glyceraldehyde-3-phosphate dehydrogenase	NM_002046	GAPDH	AAGGATAATGGCTTACAAC TCACTTAGGGCTTCTCAC	55	590

## Results

### RT-PCR Analysis

**Figure 1:**
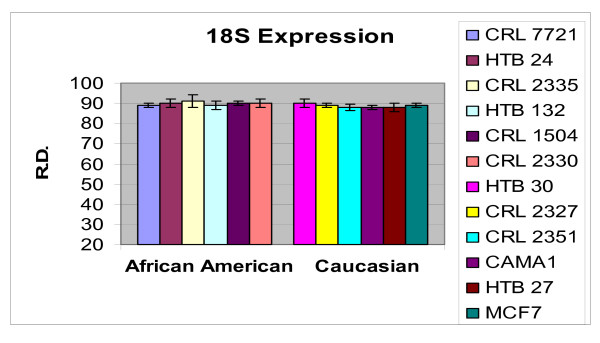
**Densitometric measurement of the 18S RT-PCR analyses**. Using the data obtained from the RT-PCR experiments, the amplicons from three different experiments of each cell line was subjected to densitometric scanning. To determine the relative density, area plots were quantitated using the Chemi Imager Tm 4000 software (Alpha Innotect, Corp. San Leandro, CA). Values from the experiments were averaged and error bars represent the standard deviation.

**Figure 2:**
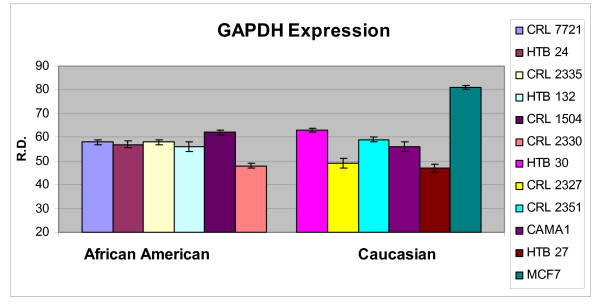
**Densitometric measurement of the GAPDH RT-PCR analyses**. Using the data obtained from the RT-PCR experiments, the amplicons from three different experiments of each cell line was subjected to densitometric scanning. To determine the relative density, area plots were quantitated using the Chemi Imager Tm 4000 software (Alpha Innotect, Corp. San Leandro, CA). Values from the experiments were averaged and error bars represent the standard deviation.

**Figure 3:**
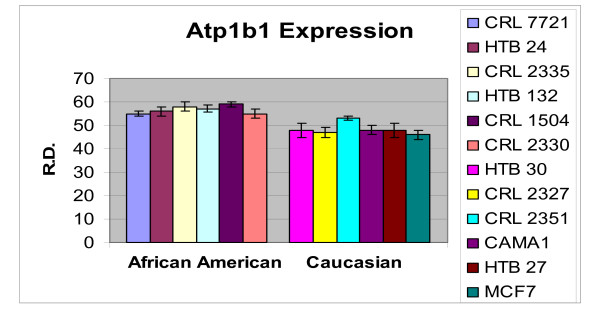
**Densitometric measurement of the Atp1b1 RT-PCR analyses**. Using the data obtained from the RT-PCR experiments, the amplicons from three different experiments of each cell line was subjected to densitometric scanning. To determine the relative density, area plots were quantitated using the Chemi Imager Tm 4000 software (Alpha Innotect, Corp. San Leandro, CA). Values from the experiments were averaged and error bars represent the standard deviation.

**Figure 4:**
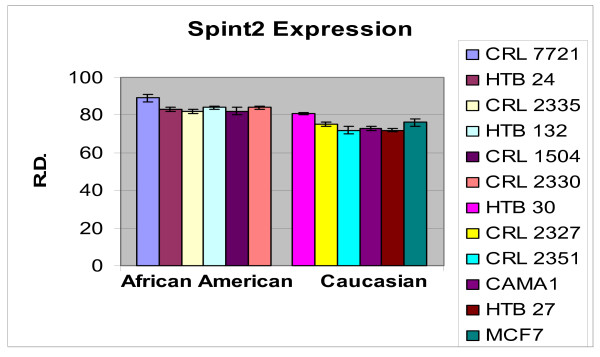
**Densitometric measurement of the SPINT2 RT-PCR analyses**. Using the data obtained from the RT-PCR experiments, the amplicons from three different experiments of each cell line was subjected to densitometric scanning. To determine the relative density, area plots were quantitated using the Chemi Imager Tm 4000 software (Alpha Innotect, Corp. San Leandro, CA). Values from the experiments were averaged and error bars represent the standard deviation.

**Figure 5:**
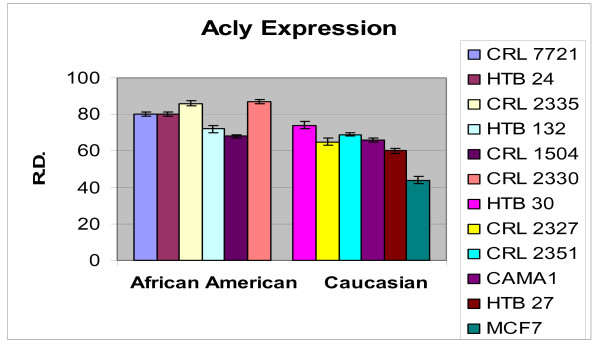
**Densitometric measurement of the Acly RT-PCR analyses**. Using the data obtained from the RT-PCR experiments, the amplicons from three different experiments of each cell line was subjected to densitometric scanning. To determine the relative density, area plots were quantitated using the Chemi Imager Tm 4000 software (Alpha Innotect, Corp. San Leandro, CA). Values from the experiments were averaged and error bars represent the standard deviation.

**Figure 6:**
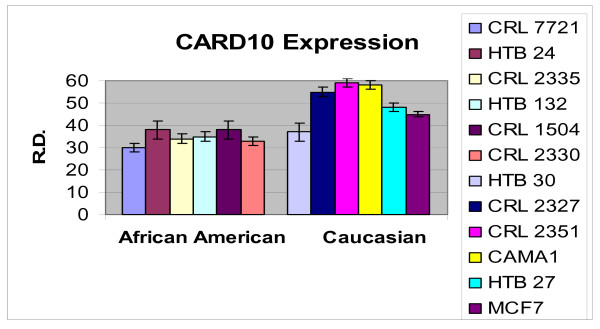
**Densitometric measurement of the CARD 10 RT-PCR analyses**. Using the data obtained from the RT-PCR experiments, the amplicons from three different experiments of each cell line was subjected to densitometric scanning. To determine the relative density, area plots were quantitated using the Chemi Imager Tm 4000 software (Alpha Innotect, Corp. San Leandro, CA). Values from the experiments were averaged and error bars represent the standard deviation.

**Figure 7:**
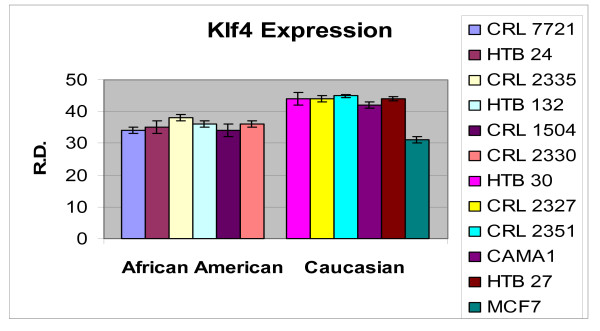
**Densitometric measurement of the Klf4 RT-PCR analyses**. Using the data obtained from the RT-PCR experiments, the amplicons from three different experiments of each cell line was subjected to densitometric scanning. To determine the relative density, area plots were quantitated using the Chemi Imager Tm 4000 software (Alpha Innotect, Corp. San Leandro, CA). Values from the experiments were averaged and error bars represent the standard deviation.

**Figure 8:**
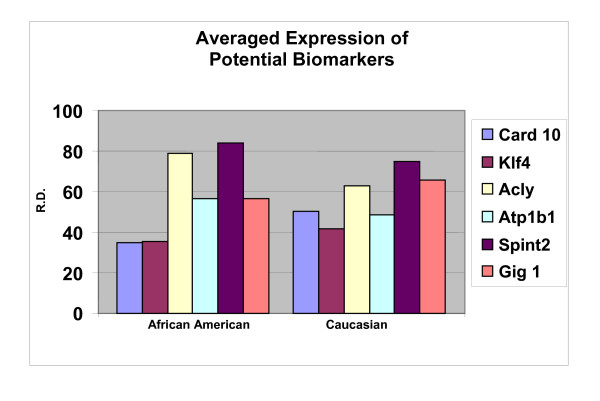
**Densitometric measurements of the RT-PCR analyses**. Using the data obtained from the RT-PCR experiments, the amplicons from three different experiments of each cell line was subjected to densitometric scanning. To determine the relative density, area plots were quantitated using the Chemi Imager Tm 4000 software (Alpha Innotect, Corp. San Leandro, CA). Values from the experiments were averaged and error bars represent the standard deviation.

In order to ascertain the cells metabolic activity and equal loading of 1 μg of RNA, expression levels of 18S and GAPDH were analyzed. The mRNA expression levels of 18S were similar between AA and CAU cell lines (Figure 1). Differential expression of the mRNA levels GAPDH was observed between AA and CAU cell lines (Figure 2) as is often seen when utilizing this internal marker. The comparison of the individual relative densities of cancer cells in both cell line models between AA and CAU women revealed altered expression in 5 of the 14 potential biomarkers (Atp1b1, CARD 10, KLF4, Spint2, and Acly) (Tables 4 and 5). Elevated expression of Atp1b1, Spint2, and Acly in AA women breast cancer cells were observed when the expression levels of all AA and CAU women cell lines were compared (Figures 3, 4, and 5). Lowered expression of KLF4 and CARD 10 were observed when the expression levels of all AA and CAU women cancer cell lines were compared (Figures 6 and 7). Altered expression of Atp1b1, CARD 10, KLF4, Spint2, and Acly in AA breast cancer cells was observed when the overall averages of the expression levels of all AA and CAU cancer cell lines were compared (Figure 8).

**Table 4:** RT-PCR expression in AA and CAU cells. Expression assessment by RT-PCR of the targeted genes using the spectrum model. Altered expression in AA vs. CAU denoted in bold. Mean ± SD

		**African American**			**Normal**		**Caucasian**		
	**Cell Lines**	**2315**	**2329**	**2320**	**AG11132**	**MCF12A**	**Hs578t**	**2336**	**MCF7**

**Gene Symbol**									
**Card 10**		**46 ± 3.17**	**41 ± 3.49**	**32 ± 0.88**	**47 ± 3.05**	**51 ± 1.46**	**45 ± 0.71**	**45 ± 2.26**	**45 ± 0.24**
**Klf4**		**29 ± 0.72**	**30 ± 0.18**	**28 ± 0.75**	**45 ± 3.70**	**35 ± 1.62**	**30 ± 0.31**	**26 ± 4.69**	**31 ± 7.49**
**Acly**		**64 ± 0.66**	**61 ± 3.80**	**78 ± 0.01**	**60 ± 5.78**	**64 ± 8.04**	**52 ± 4.76**	**50 ± 6.17**	**64 ± 0.45**
**Atp1b1**		**60 ± 2.22**	**65 ± 4.69**	**57 ± 0.35**	**61 ± 3.00**	**57 ± 0.80**	**58 ± 0.26**	**51 ± 3.40**	**46 ± 1.48**
Galnt3		37 ± 1.89	39 ± 0.40	43 ± 2.18	46 ± 0.74	56 ± 5.35	37 ± 1.37	27 ± 0.21	45 ± 1.06
Ppic		43 ± 0.76	42 ± 2.34	55 ± 0.56	46 ± 0.10	33 ± 1.60	44 ± 0.95	36 ± 2.01	43 ± 1.80
Ephx1		79 ± 4.67	67 ± 5.00	69 ± 6.29	65 ± 5.51	69 ± 6.20	73 ± 6.10	69 ± 1.18	71 ± 1.89
**Spint2**		**60 ± 0.48**	**58 ± 2.24**	**42 ± 0.10**	**66 ± 6.79**	**69 ± 0.28**	**58 ± 0.58**	**60 ± 0.11**	**56 ± 1.39**
Ptprf		32 ± 0.34	33 ± 0.15	35 ± 0.02	32 ± 0.33	27 ± 0.67	32 ± 0.01	58 ± 1.87	31 ± 0.08
Tgfb2		34 ± 0.55	37 ± 0.35	40 ± 3.22	35 ± 3.07	27 ± 0.58	42 ± 3.19	58 ± 5.44	32 ± 0.46
TIMP1		67 ± 2.10	64 ± 3.32	77 ± 3.48	57 ± 2.51	52 ± 6.11	47 ± 5.15	61 ± 2.22	74 ± 5.82
TIMP2		46 ± 4.79	66 ± 6.15	64 ± 6.95	57 ± 2.46	58 ± 8.11	48 ± 7.73	54 ± 1.16	64 ± 7.11
TIMP3		60 ± 0.08	65 ± 0.22	69 ± 0.76	56 ± 0.16	51 ± 0.22	52 ± 0.14	56 ± 0.37	76 ± 0.08
TIMP4		61 ± 0.39	63 ± 1.02	76 ± 3.38	63 ± 0.96	68 ± 7.29	77 ± 1.33	72 ± 0.16	75 ± 5.24
18S		90 ± 0.28	91 ± 0.60	89 ± 4.14	91 ± 1.75	89 ± 2.50	90 ± 1.80	89 ± 0.15	89 ± 4.98
GAPDH		83 ± 6.83	77 ± 2.34	76 ± 3.29	76 ± 0.91	75 ± 4.21	79 ± 1.55	80 ± 3.29	81 ± 7.70

**Table 5:** RT-PCR expression of MMPs in AA and CAU cells. Expression assessment by RT-PCR of the targeted genes using the metastasis model. Altered expression in AA vs. CAU denoted in bold. Mean ± SD

		**African American**						**Caucasian**					
	**Cell Lines**	**CRL 7721**	**HTB 24**	**CRL 2335**	**HTB 132**	**CRL 1504**	**CRL 2330**	**HTB 30**	**CRL 2327**	**CRL 2351**	**CAMA1**	**HTB 27**	**MCF7**

**Gene Symbol**													
**Card 10**		**30 ± 1.08**	**38 ± 2.60**	**34 ± 2.55**	**35 ± 1.56**	**38 ± 2.60**	**33 ± 0.97**	**47 ± 0.02**	**55 ± 2.28**	**59 ± 2.72**	**58 ± 0.98**	**48 ± 1.43**	**45 ± 0.24**
**Klf4**		**34 ± 0.88**	**35 ± 1.60**	**38 ± 0.99**	**36 ± 1.83**	**34 ± 0.55**	**36 ± 0.06**	**44 ± 0.88**	**44 ± 0.38**	**45 ± 0.70**	**42 ± 0.47**	**44 ± 0.85**	**31 ± 1.49**
**Acly**		**80 ± 1.43**	**80 ± 2.08**	**86 ± 1.08**	**72 ± 0.006**	**68 ± 0.25**	**87 ± 0.64**	**64 ± 2.49**	**65 ± 2.11**	**69 ± 2.27**	**66 ± 2.11**	**60 ± 2.77**	**44 ± 0.45**
**Atp1b1**		**55 ± 0.39**	**56 ± 0.31**	**58 ± 1.02**	**57 ± 0.75**	**59 ± 0.02**	**55 ± 0.39**	**48 ± 1.07**	**47 ± 1.57**	**53 ± 1.41**	**48 ± 0.32**	**48 ± 2.51**	**46 ± 1.48**
Galnt3		43 ± 2.30	42 ± 4.41	55 ± 0.41	60 ± 0.93	61 ± 1.57	49 ± 4.00	48 ± 0.65	57 ± 5.22	52 ± 1.47	53 ± 5.12	60 ± 1.49	45 ± 1.06
Ppic		49 ± 0.24	57 ± 1.02	59 ± 1.38	57 ± 0.45	61 ± 2.66	55 ± 2.46	57 ± 1.53	62 ± 1.01	62 ± 0.16	61 ± 2.71	59 ± 1.02	43 ± 1.80
Ephx1		48 ± 1.08	70 ± 0.06	63 ± 0.33	40 ± 0.52	38 ± 0.91	61 ± 1.08	53 ± 4.66	60 ± 1.16	66 ± 4.47	54 ± 0.88	72 ± 5.21	51 ± 1.89
**Spint2**		**49 ± 2.77**	**43 ± 0.96**	**42 ± 0.70**	**54 ± 0.92**	**52 ± 0.11**	**44 ± 1.14**	**56 ± 1.24**	**55 ± 2.29**	**52 ± 2.76**	**53 ± 1.17**	**52 ± 0.76**	**56 ± 1.39**
Ptprf		44 ± 1.27	40 ± 0.15	51 ± 3.00	45 ± 3.48	57 ± 3.25	52 ± 1.23	44 ± 1.90	48 ± 0.88	58 ± 0.87	55 ± 1.24	57 ± 2.89	31 ± 0.08
Tgfb2		34 ± 2.23	42 ± 0.55	39 ± 1.84	47 ± 4.51	43 ± 2.00	37 ± 1.16	33 ± 0.29	29 ± 1.95	39 ± 1.12	51 ± 4.43	34 ± 0.33	32 ± 0.46
TIMP1		68 ± 2.13	69 ± 0.31	63 ± 0.48	60 ± 1.30	67 ± 3.56	60 ± 1.30	84 ± 0.99	75 ± 4.73	59 ± 0.16	57 ± 1.18	56 ± 3.34	74 ± 5.82
TIMP2		59 ± 0.29	61 ± 3.43	62 ± 8.27	58 ± 0.44	64 ± 1.95	66 ± 4.45	64 ± 1.95	64 ± 0.77	61 ± 0.38	70 ± 5.21	53 ± 6.83	64 ± 7.11
TIMP3		83 ± 0.07	87 ± 0.09	62 ± 4.52	62 ± 3.93	84 ± 4.70	87 ± 0.75	57 ± 0.14	93 ± 1.14	77 ± 2.29	61 ± 1.17	83 ± 0.27	76 ± 0.08
TIMP4		70 ± 1.72	70 ± 5.23	69 ± 3.92	77 ± 1.65	79 ± 1.74	79 ± 1.07	64 ± 4.54	72 ± 0.43	66 ± 4.14	76 ± 2.18	73 ± 0.41	75 ± 5.24
18S		89 ± 0.98	90 ± 1.23	91 ± 1.19	89 ± 0.44	90 ± 2.22	90 ± 3.01	90 ± 0.67	89 ± 0.43	88 ± 1.51	88 ± 1.82	88 ± 1.73	89 ± 4.98
GAPDH		58 ± 1.34	57 ± 0.78	58 ± 2.12	56 ± 0.59	62 ± 0.28	48 ± 1.25	63 ± 0.71	49 ± 1.57	59 ± 1.19	56 ± 1.34	47 ± 0.41	81 ± 7.70

## Discussion

Breast cancer is described as heterogeneous because it is a different disease in different women, a different disease in different age groups and has different cell populations within the tumor itself. It is the most frequently diagnosed cancer in women. The main types of breast cancer are ductal carcinoma in situ, invasive ductal carcinoma, lobular carcinoma in situ, invasive lobular carcinoma, medullary carcinoma, and Paget's disease of the nipple. Carcinomas are malignant tumors that begin in the lining layers of organs [13].

There are many factors that contribute to breast cancer development. The risk of being diagnosed increases as individuals' age. The primary factors that increase the risk are an inherited mutation of the BRCA1 and/or BRCA 2 genes, a family history of breast cancer, high breast density, and a confirmed biopsy of hyperplasia. Although the BRCA mutations are one of the primary factors for developing breast cancer, it accounts for only 5% of all breast cancer cases. Women that carry these mutations, however, have a lifetime risk of developing breast cancer ranging from 35% to 85%. There are other factors that have been linked to increased risk of breast cancer. These include a long menstrual history, obesity after menopause, recent use of oral contraceptives, having more than one alcoholic drink per day, and postmenopausal hormone therapy. This is especially true of the use of combined estrogen and progestin therapy [14].

This study demonstrates the validity of using the spectrum and metastasis model systems to identify expression profiles that may be unique to metastatic breast cancer in AA women. We expanded our potential biomarker gene list to include 14 genes that have been shown to play roles in cancer. We observed altered expression of 5 genes in AA cell lines when compared to their CAU counterparts. This evidence suggests that the altered expression levels of CARD10, KLF4, Acly, Atp1b1, and Spint2 may be contributing factors in the higher mortality rates of AA breast cancer patients. The candidates identified could not only be useful in early detection of metastatic potential in AA women, but also provide support for the concept that there are clearly genetic factors that play a role in the aggressive phenotype seen in this population.

The data show that the mRNA transcript encoding Na K-ATPase (Atp1b1) was elevated in the cancer cell lines compared to the normal cell lines and exhibited altered expression profiles between AA and CAU cell lines. The active form of Atp1b1 is composed of a 100–112 kDa catalytic ct-subunit and a 45–60 kDa glycoprotein 13-subunit [15]. Three different isoforms of α (α_1_, α_2 _and α_3_) and β (β_1_, β_2 _and β_3_) subunits are known [16-18]. At the aa level, α isoforms share 85% sequence similarity [16] while 50% similarity is found among β isoforms [18]. Interestingly, the expression of different isoforms seems to follow a tissue-specific pattern. The α_1 _and β_1 _isoforms are produced in most tissues, whereas α_2_, α_3 _and β_2 _are produced predominantly in the brain [17,19]. The abundance of ATP1B1 has recently been found to be a useful tool for the proper reclassification of samples as either tumors of low malignant potential or the invasive tumors of epithelial ovarian cancers [20].

We observed decreased levels of both Caspase recruitment domain family (CARD) and Kruppel-like-factor 4 (KLF4) expression in both primary and metastatic cell lines compared with the normal cell lines, with the lowest expression levels occurring in the metastatic cell lines. CARDs are protein modules found in many proteins that regulate apoptosis [21-23]. A total of 21 human CARD-containing proteins have been identified. Members of this family include caspases 1, 2, 4, 5, 9, and 13, Apaf-1, CARD4 (Nod1), Nod2, CARD7 (DEFCAP/NAC), c-IAP-1, c-IAP-2, RICK (RIP2/CARDIAK), ARC, BCL10, RAIDD, ASC, Iceberg, CARD9, CARD11, and CARD14. CARD10 is a novel CARD/MAGUK family member that signals the activation of NF-κβ through BCL10. CARD10 and other members of the CARD/MAGUK family likely play an important role in receptor-mediated activation of BCL10 and NF-κβ [24].

Krüppel, a group of zinc-finger-containing transcription factors found in *Drosophila melanogaster*, is thought to control embryogenesis [25]. Among these transcription factors, a group named Kruppel-like-factors (KLFs) has recently emerged as important contributors to the development of the mammalian embryo. The KLF family consists of at least 16 members that have been separated into subgroups based on their structure [26]. KFL4 was first identified from a human umbilical vein erythroid cDNA library. The Human KLF4 gene locus is located at chromosome 9q31, which covers a 6.3 kb region [27]. KLF4 has been found to be important in regulating the proliferation and differentiation of specific epithelial and endothelial tissues [28]. The significant loss of KLF4 has been reported in sporadic colonic adenomas and carcinomas [29], gastric cancers [30], bladder cell lines and tissue [31], and lung cancer [32]. The expression of KLF4 has been found to be significantly repressed in human gliomas-associated vascular endothelial cells as compared with that found in non-neoplastic control vascular endothelial cells [33], suggesting that KLF4 is involved in an anti-angiogenic pathway.

Elevated expression of Serine protease inhibitor, Kunitz type 2 (SPINT2)/Hepatocyte growth factor activator inhibitor type 2 (HAI-2) (SPINT2/HAI-2) in both primary and metastatic cell lines compared with the normal cell lines with the highest expression levels occurring in the metastatic cell lines. We additionally observed lowered expression in AA cell lines compared to their CAU counterparts. SPINT2/HAI-2 has a broad inhibitory spectrum and was independently reported to be a placental bikunin [34]. SPINT2/HAI-2 inhibits tissue and plasma kallikreins, trypsin, plasmin, factor XIa, and chymotrypsin. Little is known about the *in vivo *functions of SPINT2/HAI-2, but disruption of the HAI-2 gene has resulted in embryonic lethality in mice indicating a potentially important role of this inhibitor in cellular development [35]. SPINT2/HAI-2 has been found to be over-expressed in pancreatic cancer [36] and ovarian cancer [37] and has been inversely correlated with tumor progression in renal cell carcinoma [38] and breast cancer [39].

The data revealed elevated levels of ATP citrate lyase (Acly) expression in both primary and metastatic cell lines compared to the normal cell lines with the highest expression levels occurring in the metastatic cell lines. We also observed elevated expression found in AA cell lines compared to their CAU counterparts. Acly is critical for the conversion of glucose to cytosolic acetyl CoA and therefore for glucose-dependent lipogenesis. Acly deficiency in mice has resulted in embryonic lethality, with no viable embryos detectable even at early stages of development [40]. Previous studies have noted that cancers exhibit high levels of glycolysis and lipogenesis [41]. Accelerated fatty acid synthesis (FAS) in tumor tissues was first reported in the 1950s [42]. Elevated expression of FAS in malignant cells has been documented in various cancers including breast [43-47], prostate [48-51], ovarian [52], endometrial [53], colon [54], tongue [55], lung carcinoma, [56] and hepatocellular carcinoma [57].

In conclusion, we have amassed two cell line models that could be used to identify biomarkers and treatments unique to AA women with breast cancer. The significance of the study is that Atp1b1, CARD 10, KLF4, Spint2, and Acly are not only useful in serving as possible biomarkers of metastasis, but also provide support for the concept that they could serve specifically as biomarkers for AA women. To date, this is the only study that has used an AA model system specifically for gene mining to investigate cancer and metastatic gene expression in cell lines derived from African American patients.

## Abbreviations

AA-African American women, CAU-Caucasian, RT-PCR-Reverse transcriptase Polymerase Chain Reaction, ECM-Extracellular matrix, MMP-Matrix Metalloproteinase

## Competing interests

The author(s) declare that they have no competing interests.

## Authors' contributions

HFY conceived the study and participated in its design, was responsible for primer design, performed data analysis, densitometric readings of RT-PCR, and prepared the first draft of the manuscript. JAM participated in the study design, was responsible for primer design, performed the RT-PCR experiments, was involved in tissue culture, and prepared the first draft of the manuscript. SP performed RT-PCR experiments, data analysis, densitometric readings of the RT-PCR, and assisted in the editing of this manuscript. CETIII performed RT-PCR experiments, data analysis and densitometric readings of the RT-PCR. GKL participated in the funding, as well as data analysis and editing of the manuscript. MJ participated in study concept, design and data analysis. AAD participated in the study concept, study design, secured funding, interpretation of the data, as well as preparation of the manuscript. All authors read and approved the final manuscript.
